# Umbilical Vein Calcification Associated with Double-Lumen Catheter Malpositioning in an Extremely Low-Birth-Weight Infant

**DOI:** 10.3390/pediatric16010007

**Published:** 2024-01-12

**Authors:** Takuya Yamamoto, Shigeo Iijima

**Affiliations:** 1 Department of Pediatrics, Hamamatsu University School of Medicine, 1-20-1 Handayama, Higashi-ku, Hamamatsu 431-3192, Japan; yamamoto1114.256@gmail.com; 2 Department of Regional Neonatal-Perinatal Medicine, Hamamatsu University School of Medicine, 1-20-1 Handayama, Higashi-ku, Hamamatsu 431-3192, Japan

**Keywords:** umbilical venous catheter, multi-lumen catheter, vascular calcification, umbilical vein, ductus venosus, extremely low-birth-weight infant

## Abstract

Umbilical venous (UV) catheters (UVCs) are commonly used in severely ill neonates. Complications associated with UVC often result from an inappropriate UVC position. Calcification of the UV, a rare complication, was observed in an extremely low-birth-weight infant born at 23 weeks of gestation. After birth, the infant experienced respiratory and circulatory dysfunction, followed by disseminated intravascular coagulation (DIC). A UVC was inserted, and circulatory agonists and blood transfusions were administered, as well as a calcium gluconate infusion for hypocalcemia and hyperkalemia. Ten days after birth, calcification was detected in the UV, likely due to a tunica intima injury caused by UVC, a hypercoagulable state due to DIC, and a high-dose calcium gluconate infusion. Additionally, proximal port malpositioning of the double-lumen catheter might have contributed to calcification within the UV. To prevent such complications, real-time ultrasound confirmation with agitated saline contrast during UVC placement is recommended; in the absence of the facility or skills for ultrasonography, X-rays should be performed in the lateral and anteroposterior views. Furthermore, when using multi-lumen catheters, physicians should not only verify the tip position but also ensure proper placement of proximal ports and carefully select medications administered through the ports.

## 1. Introduction

In fetal circulation, the umbilical vein (UV) enters the abdomen and delivers oxygenated blood primarily to the liver via the left portal vein (PV), but some of this blood bypasses the liver through the ductus venosus (DV) and enters the inferior vena cava (IVC) [1].

Since Diamond introduced intravascular catheterization in the UV for exchange transfusions in 1947 [2], UV catheterization (UVC) in severely ill neonates has remained a necessary and even life-saving neonatal intensive care procedure. Especially in extremely low-birth-weight (ELBW) infants, in whom the placement and maintenance of peripheral venous routes is difficult, a UVC is a good alternative for the provision of fluids, medications, and total parenteral nutrition, etc. Moreover, a multi-lumen approach has been adopted to reduce the need for additional venous lines for these vulnerable patients.

Despite their benefits, the use of UVCs has been associated with multiple complications, including catheter-related infections, thrombosis, intestinal perforation, hepatic necrosis, arrhythmias, myocardial perforation, and pericardial effusion [3,4]. These complications often result from inappropriate positioning of the catheter [5]. One rare but clinically significant complication associated with malpositioning of the UVC is the development of calcification within the UV. It is characterized by the deposition of calcium salts within the vessel wall, leading to structural changes that can impair blood flow, or the extravasation of calcium-containing infusions out of the injured vessel, resulting in calcinosis and/or necrosis in the surrounding tissues; both of these pose a substantial risk to the affected neonate.

In this case report, we present the clinical details of an ELBW infant who developed UV calcification as a consequence of UVC malpositioning.

## 2. Case Presentation

**Figure 1:**
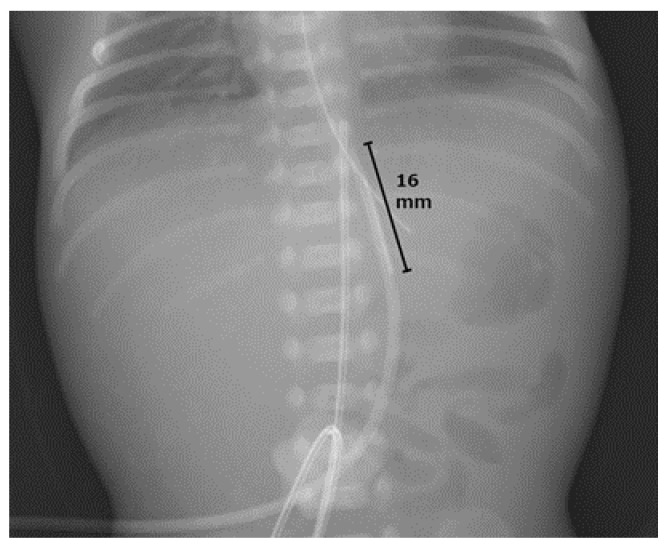
Frontal anteroposterior X-ray of the abdomen just after umbilical vein catheter placement on the day of birth. It shows the tip of the umbilical venous catheter at the Th10 level and the proximal port located 16 mm from the tip.

A male infant was born at 23 weeks and 6 days of gestation, weighing 738 g, to a 23-year-old, nulliparous primigravida, following a premature onset of labor. The mother was treated with ritodrine from 22 weeks and 2 days of gestation and magnesium sulfate at 22 weeks and 5 days of gestation to control her uterine contractions. The infant was born via spontaneous vaginal delivery, and his Apgar scores at 1 and 5 min were 1 and 2, respectively. Immediately after birth, the infant was intubated and treated with an exogenous surfactant for respiratory distress syndrome. After admission to the neonatal intensive care unit, an umbilical artery catheter (umbilical vessel catheter 3.5 Fr, 38 cm) was placed at Th7 with an 11 cm fixed tip. However, UV access was difficult to achieve, and anteroposterior (AP) X-ray images were obtained every time the catheter position was corrected. Thereafter, a UVC (4 Fr × 13 cm ARROW Pediatric Two-Lumen Central Venous Catheter; Teleflex Medical, Japan) was placed at Th10 with a 5 cm tip (Figure 1).

**Figure 2:**
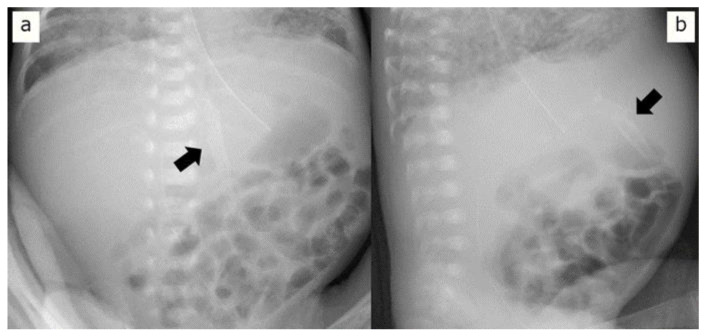
X-ray images 11 days after birth. (**a**) Frontal anteroposterior X-ray shows tram-track calcifications in the left upper quadrant (arrow). (**b**) Lateral cross-table view of the abdominal X-ray shows the calcification involving the umbilical vein (arrow).

**Figure 3:**
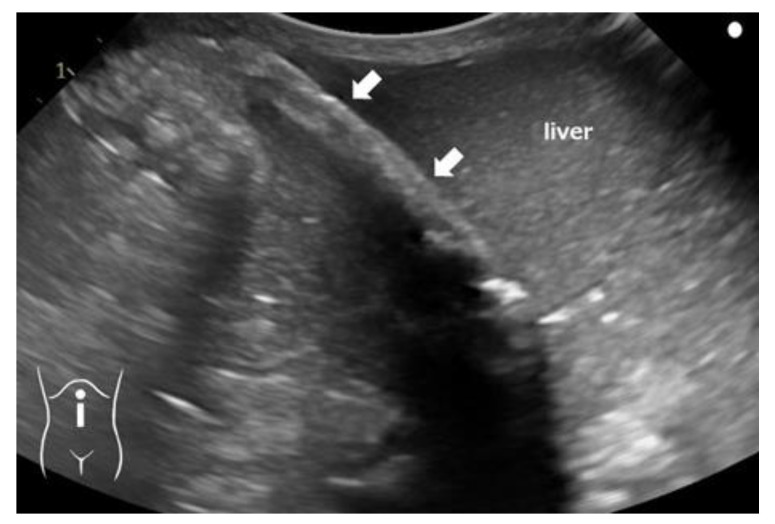
Longitudinal ultrasonographic image of the upper abdomen 11 days after birth. It shows an echogenic linear focus with posterior shadowing in the region of the umbilical vein (arrows).

Four hours after birth, the patient experienced circulatory collapse with persistent pulmonary hypertension and disseminated intravascular coagulation (DIC). He was treated with dopamine, dobutamine, hydrocortisone, nitric oxide inhalation, and transfusions of packed red blood cells and fresh frozen plasma. Continuous intravenous administration of 8.5% calcium gluconate (containing 7.85 mg/mL elemental calcium) was initiated at 8 mL/kg/day via the proximal lumen. For severe hypocalcemia and hyperkalemia, the dose was temporarily increased to 10 mL/kg/day [6]. Subsequently, the dose was maintained at 6 mL/kg/day. Ten days after birth, the infant developed a fungal infection; the catheter was suspected to be the source of infection and was removed. The next day, a plaque-like radio-dense area on the left side of the abdomen was detected on an abdominal X-ray image (Figure 2a). In the cross-table lateral view, the position and shape of the area suggested that it was located in a part of the round ligament of the liver (Figure 2b). Ultrasonography (US) revealed a hyperechogenic structure with a posterior acoustic shadow along the course of the UV remnant (Figure 3). Moreover, the blood flow revealed by the US was not indicative of patent DV. These discoveries indicated the presence of calcifications in the UV. No lesion-associated symptoms were observed during the subsequent disease course. At 80 days of age, no changes were observed in the calcified lesion upon radiography or US. At the age of 8 months, the calcified lesion in the abdomen could still be detected by US, although it was no longer radiologically detectable.

## 3. Discussion

Calcification associated with UVC is commonly observed in the liver [6,7,8,9]. However, regarding UVC-related calcification in the DV or UV, only two reports were previously published [7,8]. Rizzo et al. reported on calcification of the DV with a curvilinear appearance on X-ray images [7], caused by catheter-induced intimal injury and extravasation of calcium gluconate. Schneider et al. reported on calcification of the UV and intrahepatic branches of the PV that developed in a neonate following UVC [8] and explained that the hypercoagulable state due to bacterial sepsis resulted in thrombosis of these vessels, with subsequent calcification and extension of thrombosis from the UV into the PV system, facilitated by catheter malpositioning. Even when the physicians are highly skilled, use soft catheters, and minimize the duration of UVC, trauma to the tunica intima may occur, which may lead to thrombus formation [9].

In the present case, as well as in the abovementioned reports, the hypercoagulable state due to DIC and high-dose calcium gluconate infusion might have caused the thrombosis and calcification of the vessels injured by UVC, and malpositioning of the UVC might have resulted in calcification of the UV. However, upon imaging or based on the etiology, we could not distinguish whether the calcified lesions were simple calcium deposits on the vessels or calcified thrombotic vessels. UV calcification may lead to difficulty in removing the UVC [10]. If the calcification spreads to the DV, portosystemic shunts due to patent DV can lead to multi-system damage, such as cholestasis, pulmonary hypertension, and encephalopathy [11]. UVC malpositioning is reportedly associated with necrotizing enterocolitis in preterm infants [12]. Furthermore, if the catheter tip is in the umbilical recess, entry of hypertonic fluid, with a high concentration of calcium, into the portal system and fluid extravasation may cause calcification in or parenchymal damage to the liver [13], leading to portal hypertension, liver necrosis, and liver cirrhosis in the middle to long term [8]. During the patient’s clinical course, none of the abovementioned complications were identified.

The most appropriate UVC tip position is considered to be that at the junction of the IVC and right atrium (RA), or near the RA [5], just above the diaphragm. This position appears associated with the lowest incidence of complications. However, the optimal UVC position is difficult to establish because the catheter is inserted without imaging guidance. Thus, the catheter tip is prone to postcatheterization displacement or even misalignment [14], which may result in complications. To address this issue, several formulas and charts have been proposed to estimate the correct position of the UVC by using various body measurements (Table 1) [15,16,17,18]. The expected length of catheter to be inserted was calculated before insertion by using these formulas or charts. However, these methods are reportedly associated with significant failure rates [9,19,20]. As the insertion length alone may not be sufficient to determine whether the catheter tip is in the proper position, bedside X-ray imaging is required for positioning after conventional UVC [21]. In the present case, radiography after UVC insertion revealed the catheter tip at the Th10 level (just on the diaphragm). The region including the preferred tip position reportedly corresponds to the Th9–Th10 range on AP abdomen-chest radiographs [9]. Moreover, Sanders described that AP and lateral X-rays indicate the normal course and position of the UVC and that the area between Th10 and L1 corresponds to that just before the catheter enters the DV [1]. Therefore, we deemed the catheter to be inserted up to the proper position, although we performed radiography in only the AP view.

**Figure 4:**
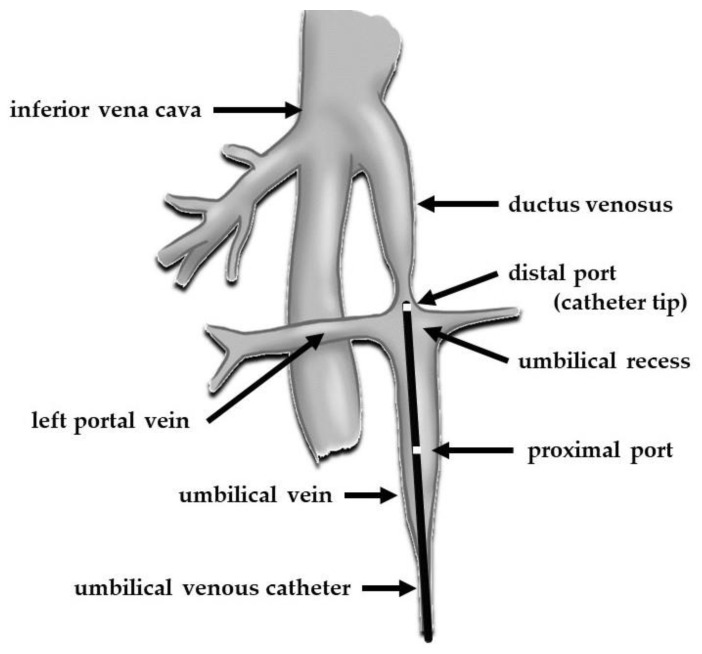
Schematic drawing demonstrating the normal anatomy of the umbilical vessels and their connections to the systemic circulation and the speculated position of the umbilical venous catheter in the present case.

At the time of insertion, the UVC enters the UV, followed by the medial part of the umbilical recess, a focal dilatation of the UV just before its junction with the left PV. Thereafter, the catheter enters the DV, eventually reaching the junction of the IVC and RA. However, the DV is narrow at its origin, with an approximate diameter of 0.9 mm at 23 weeks of gestation [22]. Additionally, functional closure of the DV occurs soon after birth. If successful, the catheter tip will pass through this narrow area and reach the IVC via a DV with a length of approximately 9 mm [22]; if unsuccessful, the catheter will stray into the left PV. In the present case, considering that the catheter was placed in an area of resistance and that further attempts at insertion were difficult, we speculated that the catheter tip was located at the inlet of the DV (Figure 4). Approximately 13–19% of UVCs do not follow the correct path towards the DV; instead, they are misaligned and follow the hepatic or portal veins into one of the hepatic lobes [23,24].

Although several reports of intrahepatic calcification caused by straying from the hepatic vein into the PV have been published [25,26,27,28], such straying can be confirmed via radiography. However, determining whether the UVC tip has passed through the DV may be difficult based on X-ray features alone. Several researchers have questioned the accuracy of X-ray features for this purpose. Hoellering et al. reported that the sensitivity, specificity, and accuracy of monitoring the catheter tip location via bedside radiography were 45%, 87%, and 66%, respectively, indicating the low reliability of this method [29]. Thus, AP X-rays alone are insufficient for the accurate and safe determination of the catheter tip location. In particular, the most inferior portion of the DV extends in the sagittal plane. In the lateral view, it can be suitably visualized [30].

Some authors advocate bedside US, which is convenient, non-invasive, and radiation-free and allows real-time guidance of UVC [31,32,33], as the gold standard for verification of the position of the UVC [34,35]. Moreover, Lafortune reported that the DV was visible upon US in the first hours after birth as a small vein coursing posteriorly and cephalically from the umbilical portion of the left PV [36]. However, US is of limited value in confirming tip placement, particularly whether the catheter tip is located in or has passed through the DV. In such cases, agitated saline contrast can help localize the tip position [37]. However, skilled personnel must be available to perform US during or immediately after UVC placement.

In the present case, the double-lumen catheter had a proximal port 16 mm from the tip, through which a high concentration of calcium was administered (Figure 4). The extension of the calcification within the UV might have been facilitated by the malpositioning of the proximal port. Although a multi-lumen approach reduces the need for additional venous lines, it may increase catheter-related malfunction [38]. The possible risks associated with the use of multi-lumen UVCs in comparison to single-lumen UVCs are blockage and thrombosis because of the narrow diameter of the internal lumen. However, previous studies have failed to address the important issue that proximal placement of multi-lumen UVCs increases the risk of injury to the tunica intima and/or extravasation from the proximal port. In adults, concerns have been raised about the risk of extravasation of infused fluid via the proximal port of multi-lumen central venous catheters if they are inserted to an inadequate depth and the side holes are not placed properly into the great veins [39,40]. In the present case, we could not precisely determine whether the catheter tip was positioned in front of or in the DV, or whether it had passed through the DV. If high-dose calcium was infused into the DV via the proximal port, it could have caused the calcification of the DV, leading to patent DV, a form of portosystemic shunt associated with subsequent complications [41]. To minimize the risk of complications associated with the port positions of the UVC, both the tip and the most proximal port must be passed a reasonable distance beyond the DV. When using a multi-lumen catheter to establish UV access, in addition to the tip position, the proximal port positions should be monitored, and care should be taken with the choice of medications administered through this port.

The main limitation of this case is that we had to place the UVC without standardized methodology in terms of its insertion and positional confirmation in a severely ill ELBW infant. A training program for the evaluation of UVC tip location and tip navigation using radiography and US should be implemented for physicians in the future.

## 4. Conclusions

In the acute management of ELBW infants, UVC is often necessary. Real-time positional confirmation by using US with agitated saline contrast is recommended to prevent UVC malpositioning; in the absence of the facility or skills for US, X-rays should be performed in the lateral and AP views to accurately identify the catheter tip position. If a multi-lumen catheter is used, attention should be paid not only to the tip position but also to the distance between the catheter tip and the most proximal port to reduce complications associated with UVC malpositioning. Standardized training programs may be necessary to prevent UVC malpositioning in the future.

## Figures and Tables

**Table 1:** Indexes to determine the appropriate insertional length of the umbilical venous catheter.

Name of Method Year and Source	Target Catheter Tip Attainment Site	Landmark or Formula for UVC Length Inserted (cm)	Estimated UVC Insertional Length for the Present Case (cm)
Dunn’s chart 1966 [15]	Between the diaphragm and right atrium	Using a specific graph based on the shoulder-umbilical length (cm)	5.0–6.5
Shukla’s formula 1986 [16]	At Th7–Th8	(BW * × 3 + 9)/2 + 1	6.6
Modified Shukla’s formula 2013 [17]	At Th8–Th9	(BW * × 3 + 9)/2	5.6
JSS formula 2019 [18]	At Th9–Th10	BW * + 6.5	7.2

* The unit of birth weight used in the calculation was kilograms. UVC, umbilical venous catheter; BW, birth weight.

## Data Availability

Data are contained within the article.
